# Sintering-Based In-Situ Synthesis and Characterization by TEM of Noble Metal Nanoparticles for Ceramic Glaze Color Control

**DOI:** 10.3390/nano11082103

**Published:** 2021-08-19

**Authors:** Karthik Lalwani, Nathan Dinh, Michael C. Leopold, Ryan H. Coppage

**Affiliations:** Department of Chemistry, Gottwald Center for the Sciences, University of Richmond, Richmond, VA 23173, USA; karthik.lalwani@richmond.edu (K.L.); nathan.dinh@richmond.edu (N.D.); mleopold@richmond.edu (M.C.L.)

**Keywords:** noble metal nanoparticles, gold salt, silver salt, ceramic glaze, oxidation, reduction, surface plasmon, coatings, color, pigments

## Abstract

Gold and silver salt mixtures are incorporated in ceramic glazes for in situ development of mixtures of gold and silver nanoparticles (NPs) that subsequently allow for a wide spectrum of low metal loading color control within ceramic materials. Prior work has shown that gold NPs can be used to create vibrant, color-rich red pigments in high-temperature ceramic and glass applications, though the achievable diameter of the gold NP ultimately limits the available range of color. The current study significantly expands color control from traditional gold nanoparticle red through silver nanoparticle green via the alteration of gold-to-silver salt ratios incorporated in the glaze formulations prior to sintering. Nanoparticle-based coloring systems are tested in both oxidative and reductive firing atmospheres. While the oxidation environment is found to be prohibitive for silver NP stability, the reductive atmosphere is able to form and sustain mixtures of gold and silver NPs across a wide color spectrum. All glazes are analyzed via reflectance spectrometry for color performance and samples are characterized via TEM and EDS for composition and sizing trends. This study creates new groundwork for a color-controlled NP system based on noble metal ratio blends that are both nontoxic and achieved with radically lower metal pigment loading than traditional glazes.

## 1. Introduction

Metallic nanoparticles (NPs) have been important in catalysis [1], biosensors [2], pharmacology [3], and artistic methodology [4,5] due to their versatility and nontoxicity [6,7] to humans. Most metallic NPs feature unique optical, electronic, and chemical properties that vary based on the NPs’ size, shape, and elemental composition [8,9]. Over the past few decades, a prominent optical property of metallic NPs that has been exploited across nearly all scientific disciplines is the surface plasmon resonance phenomena [10]. Light interacting with the NP surface will excite the outermost electrons and cause an oscillation about the metallic core, which subsequently creates an observable signal, known as the plasmon band, in the absorption spectrum. For example, gold NPs (Au-NPs) typically exhibit the plasmon band as a broadened absorption peak centered around 520 nm while Ag-NPs show a peak at 400 nm [11]. By varying the characteristics of the NP interacting with light, including aspect ratio, shape, and/or composition of the NP, the plasmon band absorption maximum can be systematically varied, resulting in solutions varying in color across the visible spectrum [11]. Solutions of spherical Au NPs, for example, exhibit shades of red while nanorods and nanocages in solution will appear blue—all due to the surface plasmon resonance effect of the different materials [12]. Solutions of pure Ag-NPs have surface plasmon band peaks at 400 nm while Au-NP solutions show the same feature at 530 nm [13].

One strategy for color control within solutions and solid matrices is to employ mixtures of NPs or NPs with alloyed centers [14]. The Lycurgus cup of the 4th century remains a remarkable and historical example of utilizing NP-color control via the mixture of gold and silver within precursor materials used to construct the cup. Indeed, TEM analysis of this Roman glass goblet reveals the apparently inadvertent inclusion of gold and silver alloyed NPs within the glass framework that allows for the cup to transition between red and green dependent on incident light [15]. More recently, the alloying of Au and Cu together also causes the absorbance peaks of Au and Cu to form an intermediate absorbance peak between 530 nm and 570 nm [16,17]. Similarly, Au-Ag alloyed NPs have been demonstrated to shift the plasmon band of the NPs, opening a wider variety of optical properties produced by Au-NPs [11,18]. While Au-NPs with diameters around 20 nm produce colors within the red spectrum, the addition of Ag blueshifts the plasmon band to produce colors in the green and blue ranges [19]. The potential tunability of the surface plasmon, and the related optical effects, have encouraged the pursuit of these color-control strategies across a wide swath of disciplines.

Recently, one of the applications of Au-NPs that seeks to exploit the surface plasmon effect is their use as an alternative colorant within ceramic glazes [4,5,20]. Ceramic glazes traditionally achieve color by incorporating heavy metals such as Pb, Cd, Co, and Cr that are toxic to humans and the environment [21]. The substitution of non-toxic Au-NPs as a colorant in ceramic glazes was shown to be resistant to surface leaching while also producing vibrant colored glazes comparable to traditional metals [4,6]. Additionally, because the plasmon effect is a dominant optical feature observable even with low concentrations of Au-NP within the glaze, it often requires at least an order of magnitude less metal (Au) than traditional heavy metals in other glazes [4]. Similarly, Ag-NPs have previously been demonstrated to produce colors in the green to light blue range in ceramic glazes, depending on their starting doping state [22]. It follows then that the incorporation of mixtures of both Au and Ag NPs, or the use of alloyed NPs, may represent strategies toward achieving greater color range and control. In addition to significantly expanding the NP-based colorant spectrum of the ceramic glaze, it also allows for improved cost effectiveness of using NPs as an alternative colorant system for ceramics. Gold remains an expensive precious metal, thereby limiting its accessibility and practicality for this particular application. The dilution of Au (~$1276 USD per ounce) content in the NPs via the substitution of Ag (~$16.44 USD per ounce) is extremely notable (nearly 8000% price difference) if the desired coloring effects can be achieved through the use of noble metal NP blends. While extensive research has been devoted to Au NP alloys, including the introduction of more affordable metals such as Ag, Al, and Cu [11,13,16,17,19,23], there are not many color systems in place that intentionally blend distinctly different NP systems for ceramic colorant control in that they must persist through the high temperatures curing process.

Even though Au-NPs have been established as an alternative coloring agent for ceramics, there are a number of complicating factors for their widespread use in this capacity. It is problematic that Au-NPs within ceramic glazes are typically fired in kilns with operating temperatures that typically exceed 1200 °C, a condition that can alter NP size/shape and subsequently affect the desired color. During cooling cycles, NPs of varying sizes can uncontrollably precipitate and alter the colorant effects as well. Both of these processes can culminate in a lack of control over desired coloring effects and subsequently restrict the versatility of NP-based coloring in ceramics. An additional issue with the use of Au-NP colorants in ceramics is that access to as-prepared Au-NPs is not ubiquitous and the continual synthesis of Au-NPs is not practical given their susceptibility toward the kiln temperatures. Similarly, the use of alloyed NPs in this manner is work-intensive, requires confirmatory characterization, and the material remains susceptible to the same problems during sintering—uncontrolled partial or complete thermal decomposition and reformation (during cooling). To circumvent these issues, an important development was the previously communicated finding that NPs could nucleate and grow from their respective metallic salts in situ (i.e., within the glaze) during kiln firing [22]. That is, the incorporation of HAuCl_4_ or AgNO_3_ within the glaze formulation prior to sintering resulted in observable Au-NPs and Ag-NPs, respectively. This phenomenon was demonstrated in varying degrees in both oxidative and reductive firing environments [22].

In this work, the simultaneous incorporation of gold and silver salt mixtures within ceramic glazes is explored as a strategy of NP-based color control not otherwise thought possible due to the size limitations of particles due to sintering. The possibility of combining the properties of red-spectrum producing Au-NPs with green-spectrum oriented Ag-NPs is shown to be a robust, versatile, and environmentally friendly colorant system that significantly expands the current catalog of NP-based ceramic glazes while also drastically reducing the cost of employing NP-based colorants in ceramics. Absorption and reflectance spectroscopy, in conjunction with transmission electron microscopy, are used to establish the methodology, formulation, and execution of an Au NP system that is then attenuated with Ag NPs for color control within ceramic glazes.

## 2. Experimental Details

### 2.1. Materials

Gold(III) chloride trihydrate (>99.9%) and silver nitrate (>99%) were obtained from Sigma Aldrich or Colonial Scientific (Richmond, VA, USA). All solutions were prepared utilizing 18.3 MΩ_cm ultrapurified water (UP H_2_O). All raw glaze materials are bulk industry standards and were purchased from ClayWorks in Richmond, VA, USA.

### 2.2. Glaze Preparation

Glaze formulations are often mixed from dry ingredients, homogenized to prevent kaolinite clumping, water is added, and they are sieved and mixed to reach a specific gravity relative to water. For each glaze sample (dry materials), a standard blend of 20% Custer feldspar, 20% Ferro Frit 3134, 20% Edgar Plastic Kaolin, 19% Flint/silica, 15% Wollastonite, and 6% Talc was used to create a well-established “clear” glaze that is functional in both midrange oxidation and high-fire reduction kilns. All Au-Ag ratio glazes were formulated with one mixture of starting dry materials and sufficient water to form a working consistency. All were sieved through a 100-mesh screen prior to addition of metal salts. To the 100:0 Au:Ag sample, 38.39 mg of HAuCl_4_ was added to the glaze mixture. Following commonly used glazing techniques, the glaze was mixed, and sample tiles were dipped in the glaze, held for three seconds, and allowed to dry before firing. To observe changes in reduction and oxidation firing kilns, samples were made in duplicate for each type of firing environment. For changing ratios of Au:Ag glazes, aliquots of AgNO_3_ were added in the following amounts by sample ratio: 100% Au: 0% Ag = 0 g of AgNO_3_; 80:20 = 0.0087 g; 60:40 = 0.0115 g; 40:60 = 0.0517 g; and 20:80 = 0.1380 g. After each aliquot of AgNO_3_ was added, the glaze solution was mixed, and the sample tiles dipped in the glaze. For the 0:100 Au:Ag glaze, a new glaze mixture was made with 0.5 g of AgNO_3_. The glazes were then fired in either reductive or oxidative kilns to cones 10 (2345 °F, 1286 °C) or 6 (2200 °F, 1200 °C), respectively, consistent with modern firing techniques.

### 2.3. Post-Firing Glaze Analysis

After firing, percent reflectance specular profiles were obtained via an Ocean Optics (Largo, FL, USA) Halogen lamp (HL-2000-FHSA) and Flame miniature spectrometer (FLAME-S-VIS-NIR-ES, 350–1000 nm) for color quantification. Additionally, this instrument was used to generate chromaticity diagrams and CIE Lab color profiles of each sample. Glaze dust was then collected in a scintillation vial via grinding with a Dremel. The dust was then processed in a mortar and pestle for roughly five minutes or until the glaze turned into a fine powder. The powder was then suspended in minimal amounts of ethanol. This process was repeated for each sample. The mortar, pestle, and Dremel drill tip were washed with acetone and water after each sample. Once all glaze samples were suspended, 5 µL of each sample was then placed on separate 400-grid copper formvar coated grids (Electron Microscopy Sciences) and imaged via TEM (JEOL 1010 with Advanced Microscopy Techniques XR-100 CCD image collection). NP diameters were then measured by pixel by standardizing the scale bar of the microscopy image using Adobe Photoshop for at least 100 nanoparticles per sample. NP elemental analysis was performed via TEM (JEOL JEM-F200 Cold FEG Electron Microscope) at Virginia Commonwealth University’s Nanomaterials Core Characterization Facility, Richmond, VA, USA (https://nano.vcu.edu/, accessed during 12–22 June 2021).

## 3. Results and Discussion

**Figure 1:**
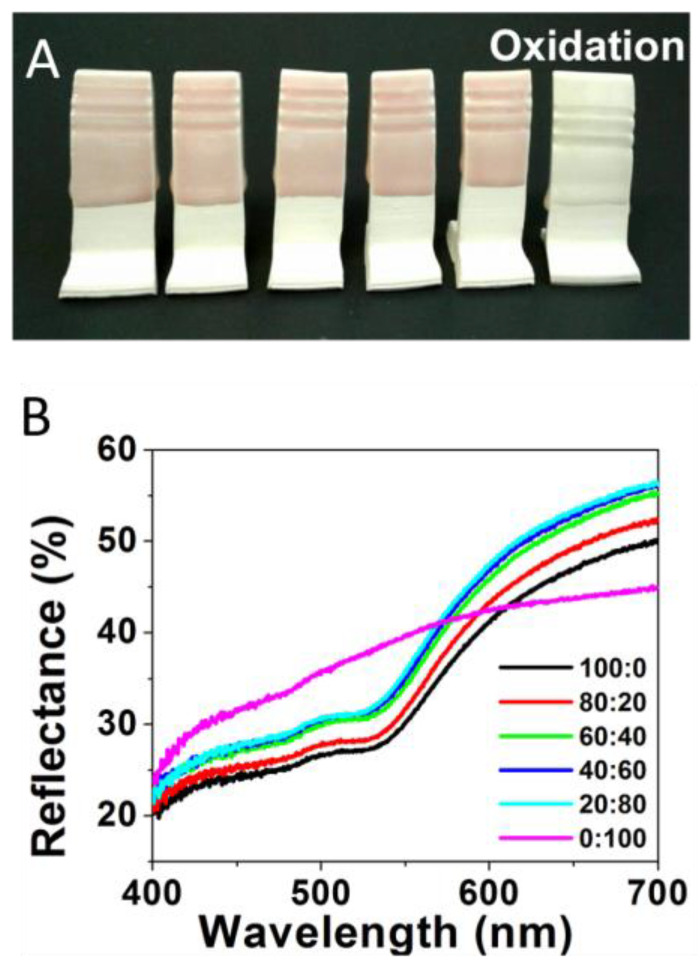
(**A**) Oxidative fired tiles formed from glaze formulations containing varying Au:Ag salt ratios of 100:0 or 100% Au); 80:20; 60:40; 40:60; 20:80; and 0:100 or 100% Ag (left to right) and (**B**) corresponding reflectance spectra for each sample.

The study was initiated by preparing standard ceramic glaze mixtures doped with different ratios of HAuCl_4_ and AgNO_3_ salts, traditional precursor salts used to form NPs in solution [4,5]. The salt ratios of HAuCl_4_ and AgNO_3_ (Au:Ag) incorporated in the glaze formulations included the following: 100:0 (100% Au), 80:20, 60:40, 40:60, 20:80, and 0:100 (100% Ag). As described in the Experimental Details section, ceramic testing tiles were immersed in the glaze mixtures containing the different salt ratios prior to being fired under oxidative conditions in the kiln. An oxidative environment was examined first as it is a more ubiquitous operational mode for amateur ceramic artists and hobbyists. Figure 1A shows the faint coloring of the glazed tiles featuring the various Au:Ag salt ratios after being fired in oxidative conditions. Only extremely subtle color changes are observed across the samples, with all the tiles exhibiting pastel pink/reddish color, a color traditionally attributed to Au-NPs [4]. The only exception is the 100% Ag sample (Figure 1A, far right), which resulted in a mostly white/clear glaze. These results demonstrate the expected limited application of silver within an oxidative firing environment (though some Ag-NP could be found via TEM) while apparently maintaining the formation of low concentrations of Au-NPs to produce a light pink shade. Metallic silver remains more susceptible to oxidation throughout the entire process.

The color trend for samples formed under oxidative conditions is reiterated upon corresponding reflectance spectroscopy measurements performed on the same tiles. Representative reflectance spectra are shown in Figure 1B. Unlike absorbance spectroscopy, a high degree of reflectance shown on a reflectance spectrum is illustrative of the transmitted or reflected wavelengths of light that mirror what a human eye would observe. As expected, reflectance spectra of the tiles from the oxidative environment, regardless of Ag content in the salt ratio, shows a relatively static change in color profiles. Chromaticity diagrams and CIE Lab color are shown in Figure S1, with the majority color peaks (for gold-containing samples) existing within 3 nm of one another—593 to 596 nm. The five samples containing any amount of Au are all relatively similar, which can clearly be seen by the lack of change in their colors. As previously mentioned, the most distinct sample is the 100% Ag sample, which has a stronger green and blue region as compared to the Au-containing samples.

**Figure 2:**
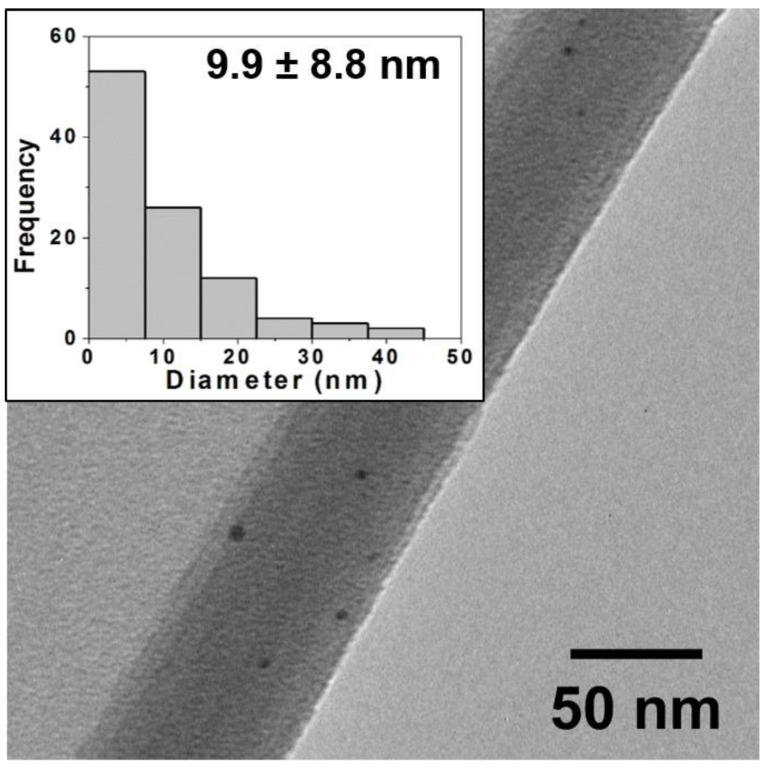
Typical TEM image for *oxidative* firing samples (80:20) and corresponding histogram analysis of core size (inset) showing a wide distribution of NP sizes with an average diameter of 9.9 ± 8.8 nm. TEM images and histograms for other ratios are provided in the Supplementary Materials. Note: NPs are often observed in images at edges of the ground ceramic shards or where the film is curled back and they are trapped as pictured above.

The brighter coloring (higher overall reflectance) of the tiles from the oxidative firing suggests a significantly low concentration of NPs in all the glaze materials is minimizing color effects derived from surface plasmon resonance effects. To ascertain the NP content within each of the glazes, each sample was ground into a fine dust and suspended in ethanol in preparation for TEM analysis (Experimental Details). Figure 2 shows a TEM image and corresponding histogram analysis of core size for the 60:40 sample that is representative of all the samples containing gold. TEM imaging of the samples indicates that the color, albeit rather muted, can be attributed to the presence of NPs formed from the salts during sintering. Additional TEM images and corresponding histogram analysis for the other ratios in the oxidation environment are provided in the Supplementary Materials (Figure S2). In examining the results from the oxidation firing, regardless of the sample, histogram analysis shows a wide distribution of core sizes (i.e., high standard deviation). However, the distribution of the 100% Au-NPs (100:0) shows a size distribution with 24.0 ± 9.7 nm average diameter Au-NPs that is then immediately skewed toward smaller diameter NPs with the introduction of any Ag content into the glaze mixture. The consistently bleak coloring of these samples is consistent with prior work that has shown the oxidative environment is less conducive to NP formation [22]. These results suggest that the necessary nucleation/growth cycle for formation of NPs from a salt precursor requires the metal cation to be reduced, which is notably challenged by the oxidative firing conditions. In this respect, TEM analysis revealing the presence of any NPs is surprising. It is hypothesized that the metal cations are reduced via a process called getter reduction, where impurities within the glaze (or kiln itself) act as sacrificial reducing agents that transfer electrons to the metal cations within the glaze as they undergo thermal degradation. The significantly higher reduction potential of Au^+3/0^ (+1.52V vs. NHE) versus that of Ag^+1/0^ (+0.80 vs. NHE) suggests that the Au^+3^ cations would be favored to be reduced over the Ag^+1^ cations, thereby facilitating Au-NP at the expense of Ag-NPs even with a mixed ratio of starting salts. These phenomena could possibly explain the lack of change in the oxidative samples containing any Ag salt until the Au salt is completely absent and eliminates the competition for free electrons. That said, the 100% Ag oxidative sample is still a pale shade of green and suggests that this mechanism and this environment results in minimal formation of all NPs but particularly diminishes formation of Ag-NPs. These results demonstrate the more limited application of Ag within an oxidative firing environment, while establishing that some formation of Au-NPs is still able to produce a light pink hue in the ceramic.

**Figure 3:**
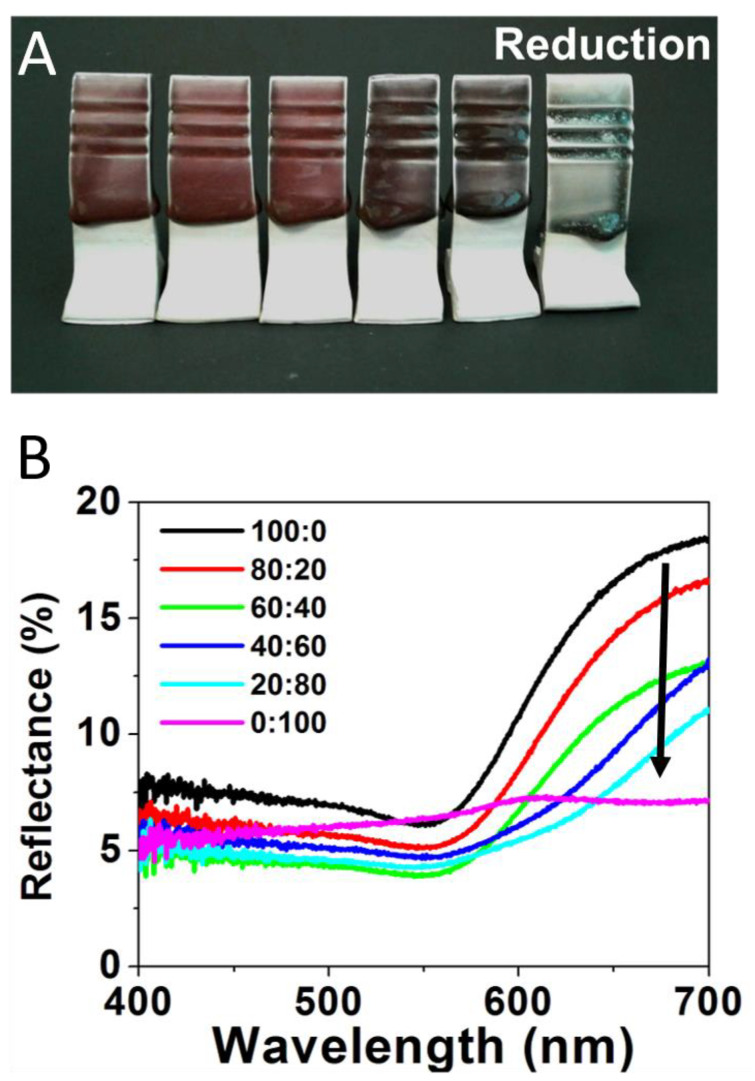
(**A**) Reductive fired tiles formed from glaze formulations containing varying Au:Ag salt ratios of 100:0 (or 100% Au); 80:20; 60:40; 40:60; 20:80; and 0:100 or 100% Ag (**left** to **right**) and (**B**) corresponding reflectance spectra for each sample.

Figure 3 repeats the same experiment utilizing a range of Au:Ag glaze mixtures for tiles fired in a reductive environment, with results showing a striking difference in the vibrancy of the color produced. In the reduction-fired samples, the 100:0 glaze mixture (Figure 3A—far left) creates a deep red color, consistent with previous reports employing Au-NPs in ceramic glazes [5,22]. The majority Au ratios (i.e., 80:20 and 60:40) maintain this general shade of red as well, but act as a brightener, yielding a more desirable red than just the pure Au-NP sample. However, upon significant introduction of the Ag content (40:60), a transition to a dark purple, which intensifies at the 20:80 ratio, is observed. The pure Ag content (0:100) glaze results in a blue-green color (Figure 3A—far right). Corresponding reflectance spectra, shown in Figure 3B, again reinforce the observed visual color trend. For these reduction samples, the gradual change in reflectance spectra directly corresponds to the systematic changes in the Au:Ag precursor salt ratios. Red wavelengths (635—700 nm) are stronger as the sample has more Au content—a property that is severely attenuated when gold is absent (0:100) and only Ag-NPs supposedly form. Accordingly, the 0:100 sample has a pronounced broad green region (520–560 nm) relative to its overall spectrum compared to the other samples. These results suggest that, in a reductive kiln, increasing amounts of silver will allow for dark yet vibrant red to green color gradients.

**Figure 4:**
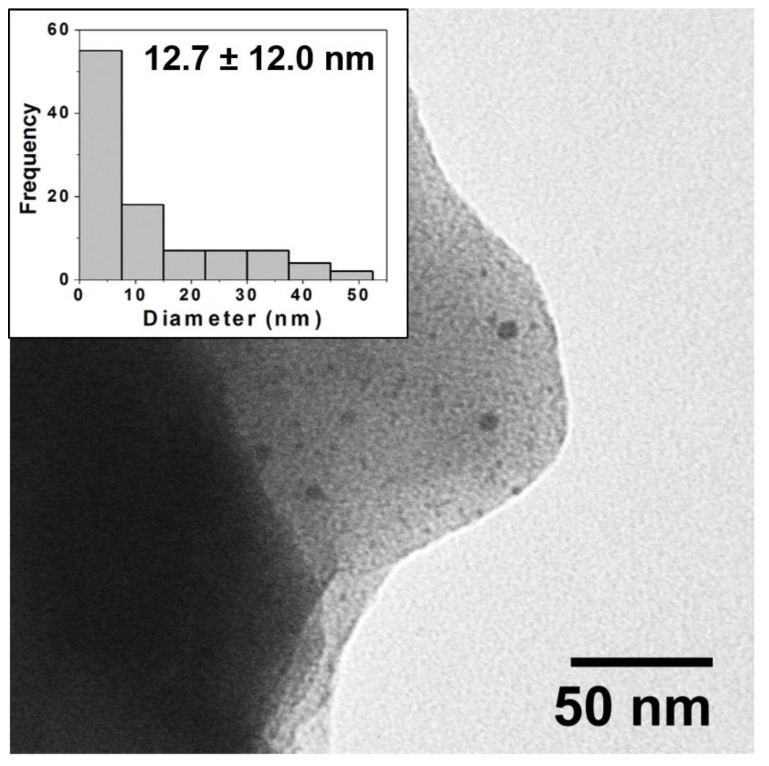
Typical TEM image for *reductive* firing samples (80:20) and corresponding histogram analysis of core size (inset) showing a wide distribution of NP sizes with an average diameter of 12.7 ± 12 nm. Note: NPs are often observed in images at edges of the ground ceramic shards as pictured above.

**Figure 5:**
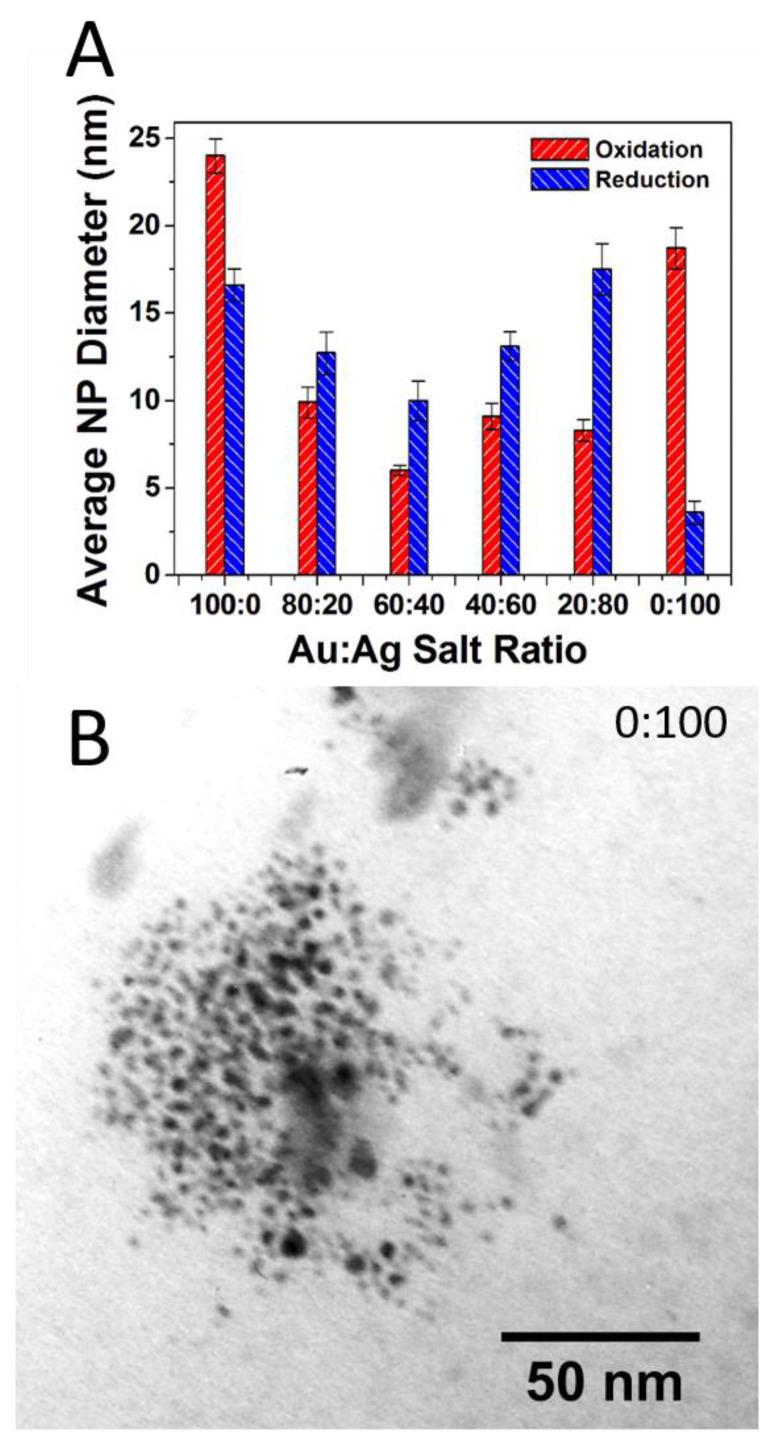
(**A**) Summary of TEM-determined average NP core size with standard deviation error as a function of Au:Ag salt ratio in the glaze formulation for both oxidative and reductive firing environments; (**B**) TEM image of reductive 0:100 or 100% Ag sample showing distinct Ag-NPs have formed during firing. Note: Standard error or σ_sample_/√n) is illustrated in Figure 5A.

Here, again, after firing TEM imaging and analysis reveals the source of the vibrant colors in the reductive samples is directly attributable to the surface plasmon effect of embedded NPs that have formed during the reductive firing. Figure 4 shows an example of a TEM image and corresponding histogram analysis for the reductive 80:20 sample with a clear indication of the presence of NPs. Again, chromaticity diagrams and CIE Lab color values were obtained (Figure S3) that show a broader range of majority color reflectance peaks (from 611 to 648 nm in the red ranges and 579 nm for the greenish 0:100 tile). Additional TEM images and corresponding histogram analysis for the other ratios in the reductive environment are provided in the Supplementary Materials (Figure S4). TEM analysis again reveals the presence of NPs through all the samples with some notable differences from the prior oxidation experiment, most notably the apparently significantly higher concentration of NPs that intensifies the surface plasmon-based color observed as well as an interesting trend in particle diameter across the different ratios. Figure 5A summarizes the average diameter of each ratio fired in both an oxidation and reductive environment. Interestingly, the average diameters are highest at 100:0 (oxidation) and 0:100 (oxidation), with smaller average diameters ranging between 12 and 18 nm observed at the mixed ratios and an incredibly small size and distribution for the 0:100 reduction sample. Unlike the 100:0 sample in the oxidative environment (Supplementary Materials—Figure S2), the 0:100 oxidative sample also shows a flat size distribution centered around relatively large diameter Ag NPs (18.7 ± 11.8 nm). TEM imaging clearly reveals the presence of NPs with only AgNO_3_ salt incorporated into the glaze formulation, suggesting the formation of Ag-NPs (Figure 5B). In the prepared 0:100 samples, there is a presence of NPs, albeit at lower incidence (concentration) compared to the pure gold sample.

**Figure 6:**
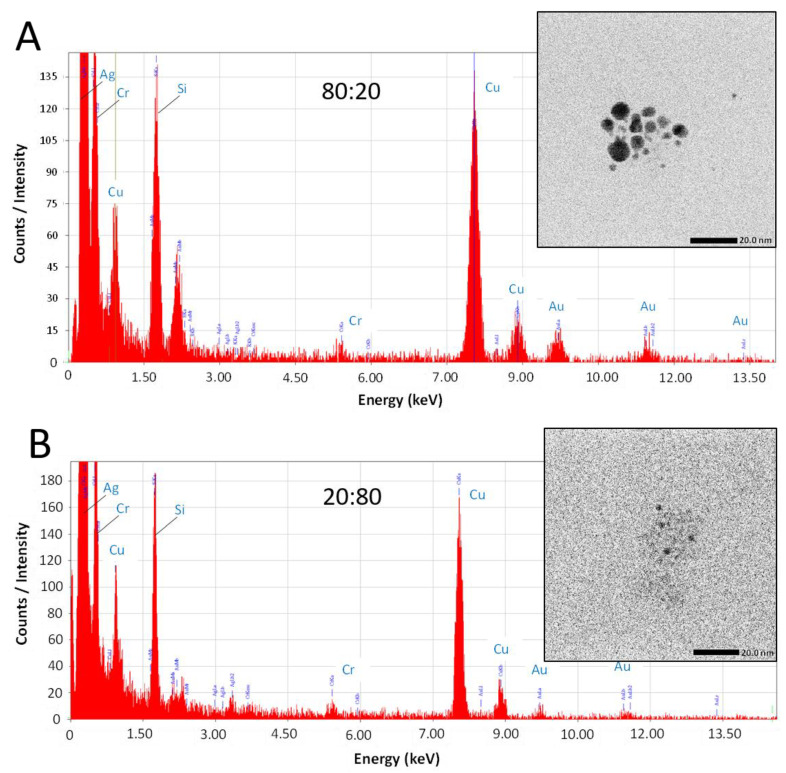
TEM imaging (insets) with EDS analysis of field with NPs for (**A**) 80:20 and (**B**) 20:80 samples fired in reductive environments.

**Figure 7:**
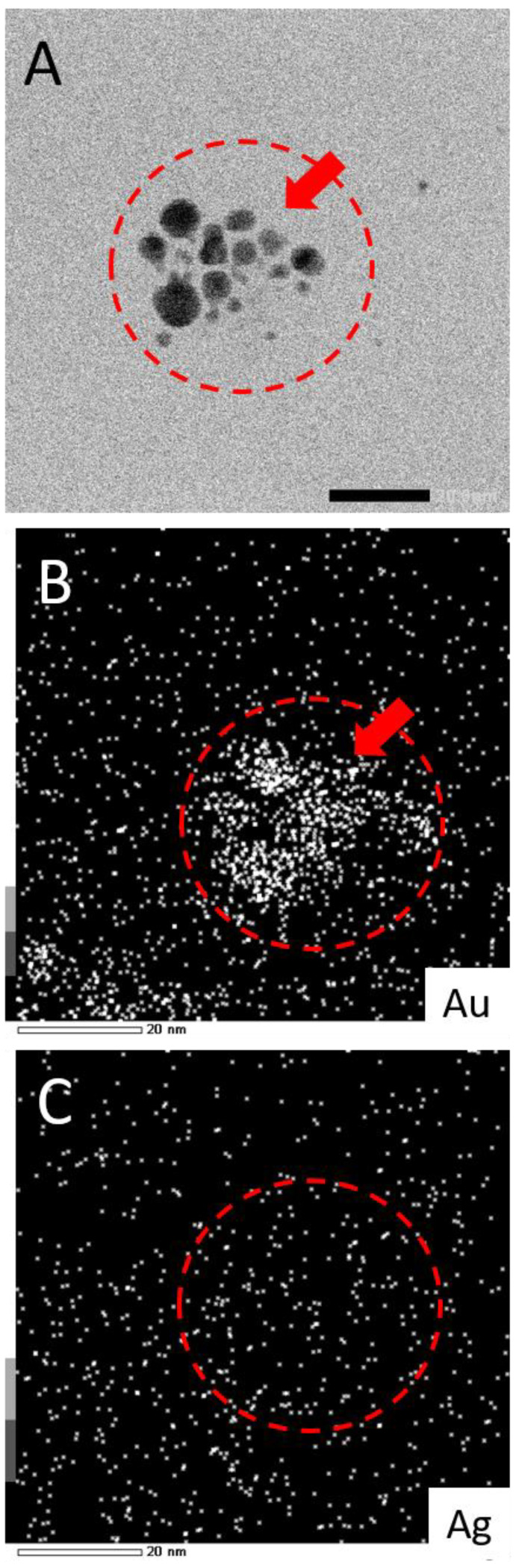
(**A**) TEM image of 80:20 reductive sample with corresponding EDS elemental mapping for (**B**) Au and (**C**) Ag. Note: Scale bar in frame A is 20.0 nm and EDS intensity scale at bottom left in frames B and C. Additional elemental mapping for Cr, Si, and Cu is shown in Supplementary Material.

While this work has established that NPs form from salts within the glaze during reductive firing, a remaining question is if the plasmonic coloring from samples using mixtures of Au and Ag salts are producing Au-Ag alloyed NPs or rather showing the collective contributions of distinct, co-existing Au-NPs and Ag-NPs in different combinations and sizes within the glaze. To address this question, select samples (80:20 and 20:80 ratios) fired in reductive environments were analyzed via TEM equipped with energy dispersive spectroscopy (EDS) for elemental analysis. Figure 6 shows representative examples of the results from this type of analysis. TEM imaging (Figure 6—insets) clearly show the presence of NPs in both samples. EDS analysis of the areas reveals typical EDS signatures for the presence of gold and silver in the scanned fields. Other signals can be attributed to the coating of the TEM imaging grid (Cu) and the non-noble metal normal impurities of the ceramic matrix (Si, Cr). Shown in Figure 7, the elemental mapping provided by the EDS of the 80:20 sample TEM image (Figure 7A) is instructive. Elemental mapping of Au for that image (Figure 7B) shows a high density of Au signal that corresponds to the same area as the imaged NPs, suggesting a major component of the NPs is Au. Elemental mapping of Ag for the same area (Figure 7C), however, shows signals evenly dispersed across the field and the absence of any high density of Ag signal. These results suggest that the 80:20 ratio results in evidence of Au-NPs and the apparent absence of Ag-NPs. That said, a similar dispersed Ag signal was found in the EDS analysis of the 0:100 or 100% Ag sample (not shown) even though in-house TEM analysis taken immediately of the sample shows the presence of Ag NPs (Figure 5B). Interestingly, when higher silver ratio (i.e., 20:80 and 0:100) samples are imaged after 48 h or more, there is little or no evidence of the same Ag-NPs in the samples (Figures S6 and S7). Given these results, it is strongly suspected that the Ag NPs, once liberated from the ceramic matrix with no stabilizing ligands, are susceptible to oxidation and subsequent degradation whereas the Au-NPs are more resistant to oxidation. Additionally, the keV (200 keV) required to perform the EDS showed live-stream disintegration of NPs, including in the 0:100 sample that would be consistent with observing a dispersed Ag signal across the field. Even with in-house TEM imaging of the 0:100 sample (80 keV), Ag-NPs were much more difficult to observe, particularly on aged samples and lighter “holes” (low electron density) were often observed with these samples (Supplementary Materials), possibly the remnants of Ag-NPs after degradation. These observations, coupled with the fact that Au-NPs persist and can be analyzed, supports the notion that the observed coloring effects and color control can be attributed to the collective presence of both Au NPs and Ag NPs present and stabilized in the glaze rather than alloyed NPs.

## 4. Conclusions

The findings of this study demonstrate that gold and silver salts at specific ratios can be incorporated within ceramic glaze formulations that, during reductive firing, promote significant nucleation and growth of Au and Ag NPs within the glaze that subsequently expand the range of available color produced. The surface plasmon effects of the embedded NPs allow for specific tuning of vibrant coloring of the glaze with the manipulation of the Au:Ag salt precursor ratio. The significant difference between the reductive and oxidative results may be explained by the reduction potentials of each metal. In a reduction kiln, the production of primarily CO is able to extract oxygen from the sample surface, leaving behind electrons and forming CO_2_. The electrons then reduce the metal to their neutral state, thus allowing for the nucleation and formation of NPs that, in turn, exhibit a minor plasmon effect for color. In the reductive environment, there is enough CO to reduce both Au and Ag, potentially facilitating the simultaneous or sequential formation of Au and or Ag NPs or mixtures of the two. As such, different combinations of metal salts in the reductive kiln are able to produce embedded NPs that exhibit a range of distinct hues. The oxidative kiln, however, has little to no production CO and, as previously explained, must rely on a notably weaker reduction mechanism toward NP nucleation that produces much less vibrant coloration.

In summary, this work has demonstrated that the use of Au and Ag salt ratios within glaze formulations provides an unprecedented level of control over the resulting NP plasmon-based colorant effects, which are more brilliant with the use of a reductive firing environment. Overall, the formation of NPs derived from salts and in situ formation represents an alternative colorant strategy in ceramics that is practical, affordable, and environmentally friendly.

## Supplementary Materials

The following are available online at https://www.mdpi.com/article/10.3390/nano11082103/s1, Figure S1: Chromaticity diagrams and CIE lab color of all oxidation-fired samples, Figure S2: TEM imaging and histogram analysis for all salt ratios in oxidation-fired samples, Figure S3: Chromaticity diagrams and CIE lab color of all reduction-fired samples, Figure S4: TEM imaging and histogram analysis for all salt ratios in reduction firing, Figure S5: TEM-EDS elemental mapping of 80:20 reduction sample, Figure S6: In-house TEM imaging of predominant silver 20:80 oxidation sample after 48+ h and Figure S7: 0:100 reduction samples immediately and after > 48 h.
