# Supplementary material for: Sintering-Based In-Situ Synthesis and Characterization by TEM of Noble Metal Nanoparticles for Ceramic Glaze Color Control
Source: Nanomaterials (Basel). 2021 Aug 19;11(8):2103. doi: 10.3390/nano11082103 (PMC8401758; doi:10.3390/nano11082103)
Supplement: Supplementary file 1 [file nanomaterials-11-02103-s001.zip › nanomaterials-1323409-supplementary.pdf]

nanomaterials

MDPI

Supplementary Materials

Sintering-Based In-Situ Synthesis and Characterization by TEM of Noble Metal Nanoparticles for Ceramic Glaze Color Control

Karthik Lalwani, Nathan Dinh, Michael C. Leopold and Ryan H. Coppage *

Department of Chemistry, Gottwald Center for the Sciences, University of Richmond, Richmond, VA 23173, USA; karthik.lalwani@richmond.edu (K.L.); nathan.dinh@richmond.edu (N.D.); mleopold@richmond.edu (M.C.L.)

* Correspondence: rcoppage@richmond.edu

Contents:

- **Figure S1**. Chromaticity diagrams and CIE Lab color measurements for the 100:0 (a), 80:20 (b), 60:40 (c), 40:60 (d), 20:80 (e), and 0:100 (f) oxidation samples.
- **Figure S2.** TEM imaging and histogram analysis for all salt ratios of glaze fired in oxidative environment.
- **Figure S3.** Chromaticity diagrams and CIE Lab color measurements for the 100:0 (a), 80:20 (b), 60:40 (c), 40:60 (d), 20:80 (e), and 0:100 (f) reduction samples.
- **Figure S4.** TEM imaging and histogram analysis for all salt ratios of glaze fired in reductive environment.
- **Figure S5.** TEM-EDS elemental mapping of 80:20 reduction sample.
- **Figure S6.** In-house TEM imaging of predominant silver 20:80 oxidation sample (Figure S4) after 48+ hours.
- **Figure S7.** In-house TEM imaging of 0:100 reduction sample immediately versus after 48 + hours.

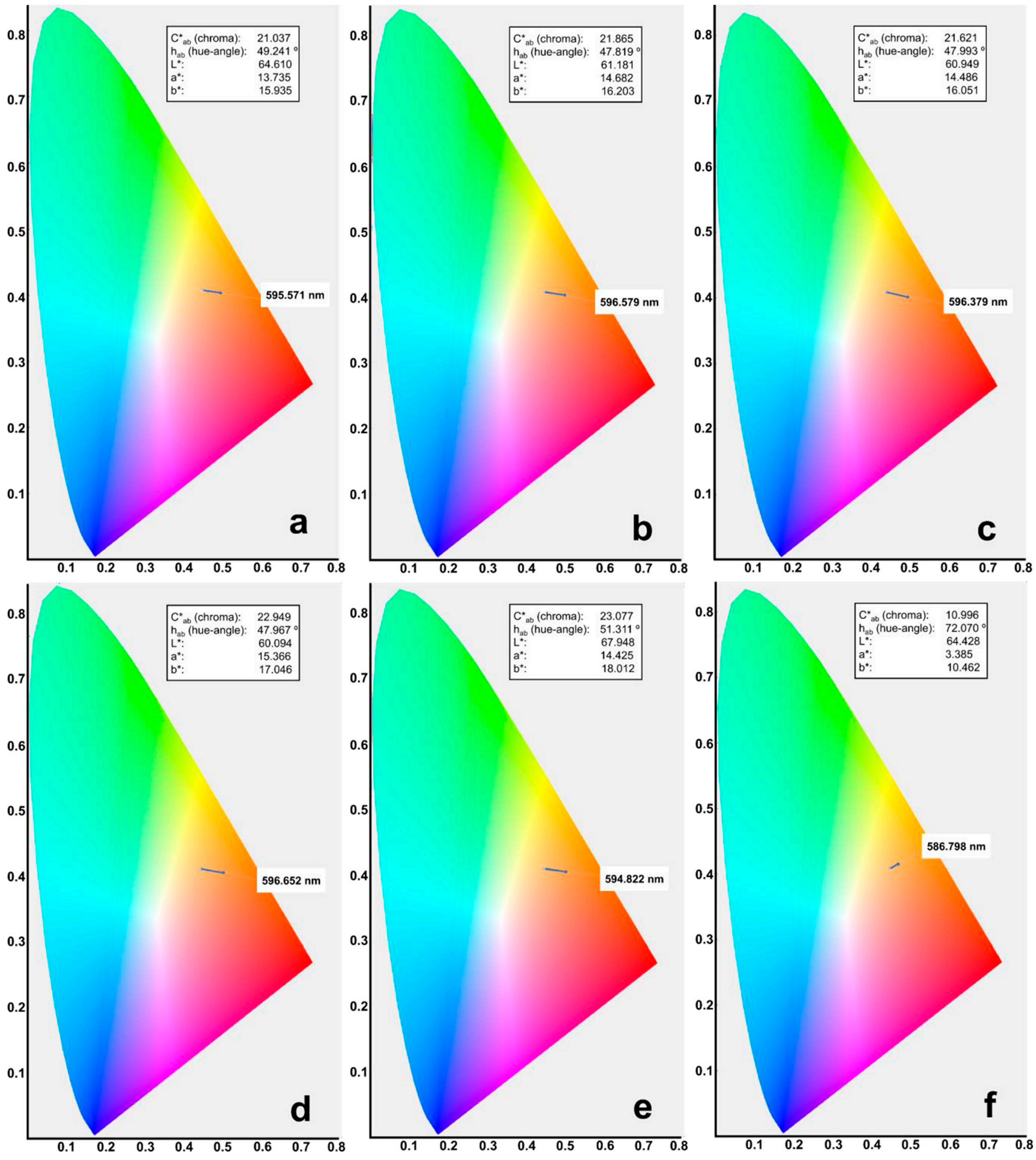

Figure S1. Chromaticity diagrams and CIE Lab color measurements for the 100:0 (a), 80:20 (b), 60:40 (c), 40:60 (d), 20:80 (e), and 0:100 (f) oxidation samples.

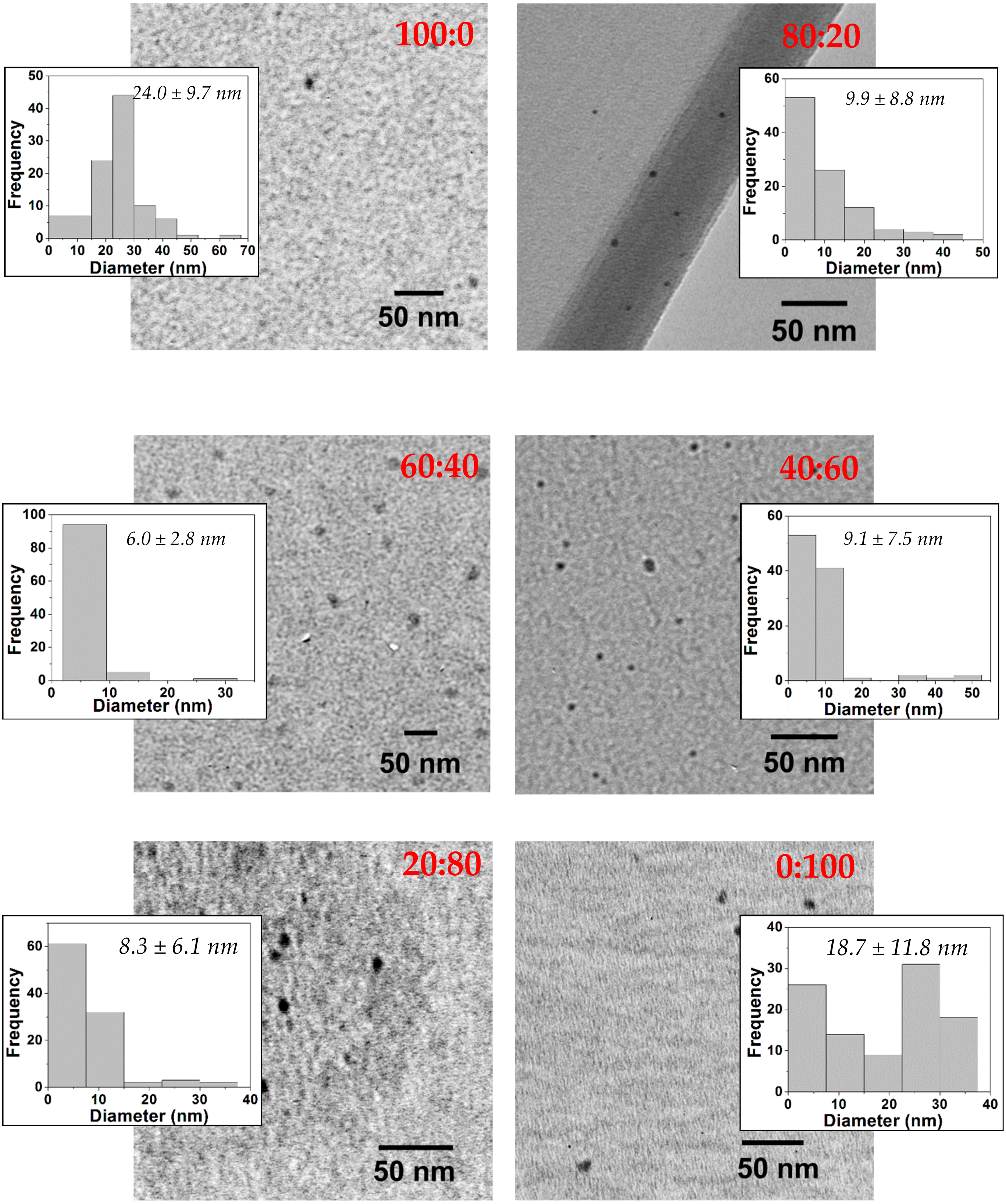

Figure S2. Typical TEM images for **oxidative firing** samples with varying Au:Ag salt ratios and corresponding histogram analysis of average core size with standard deviation (insets).

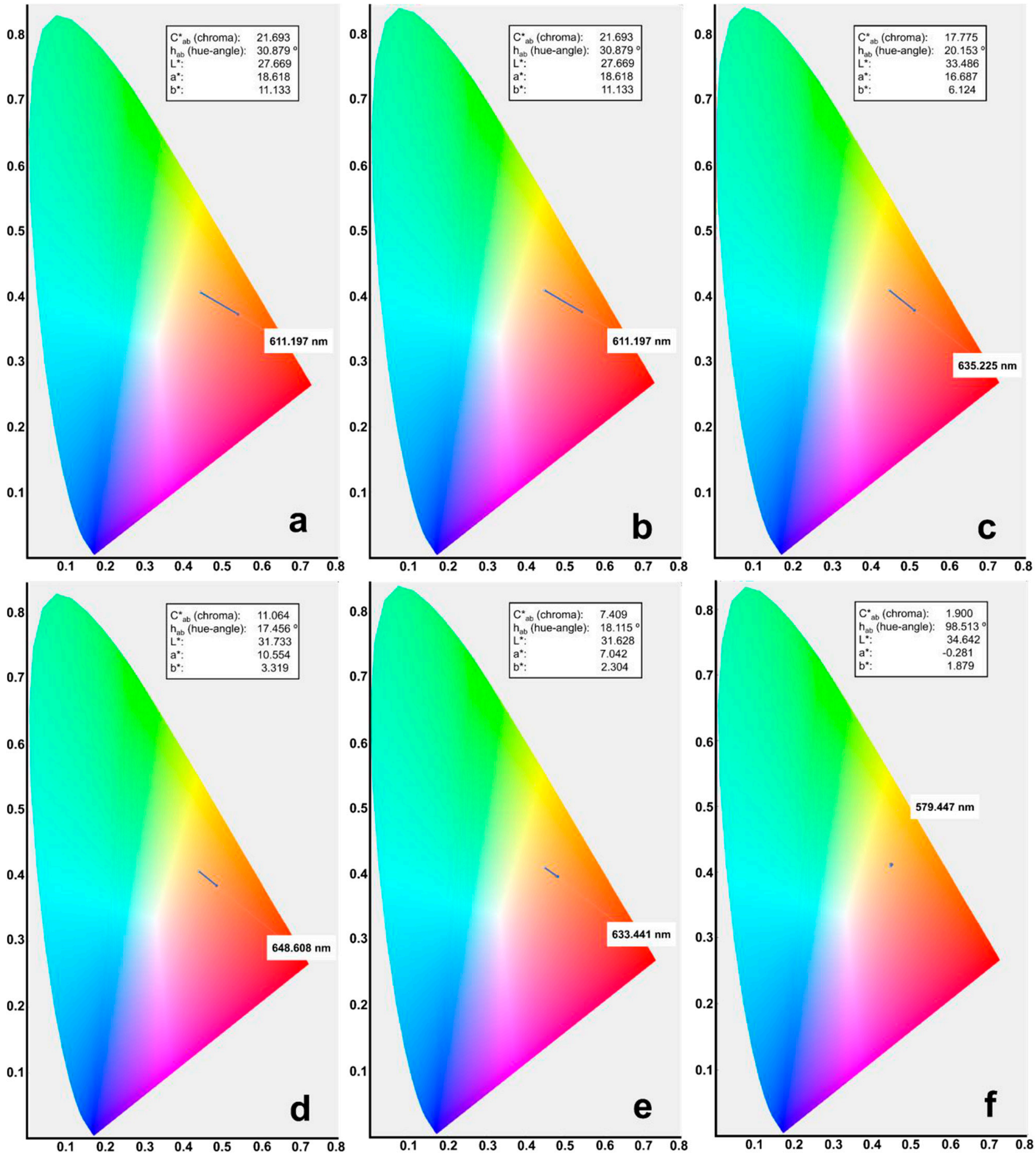

Figure S3. Chromaticity diagrams and CIE Lab color measurements for the 100:0 (a), 80:20 (b), 60:40 (c), 40:60 (d), 20:80 (e), and 0:100 (f) reduction samples.

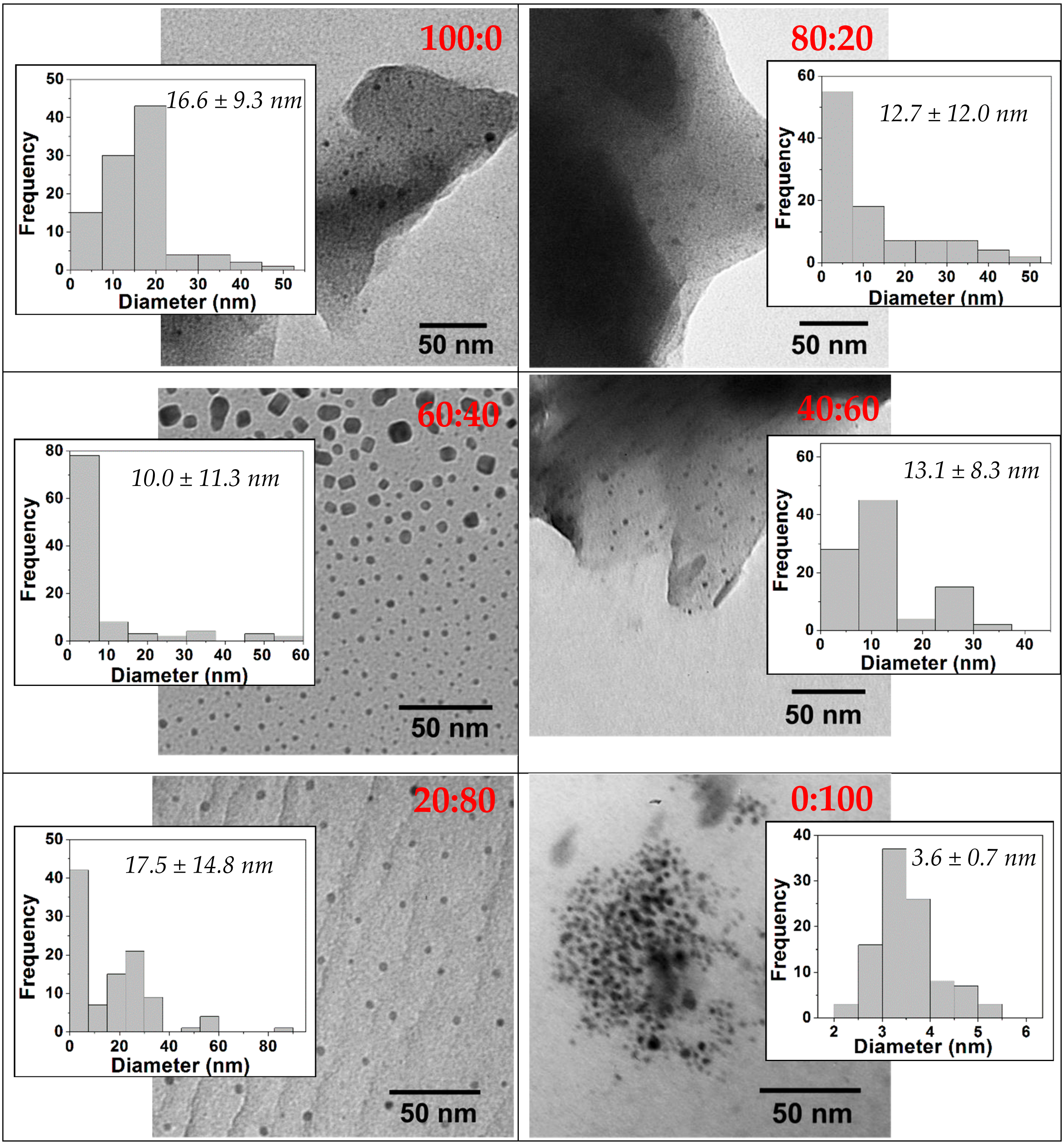

Figure S4. Typical TEM images for **reductive firing** samples with varying Au:Ag salt ratios and corresponding histogram analysis of average core size with standard deviation (*insets*).

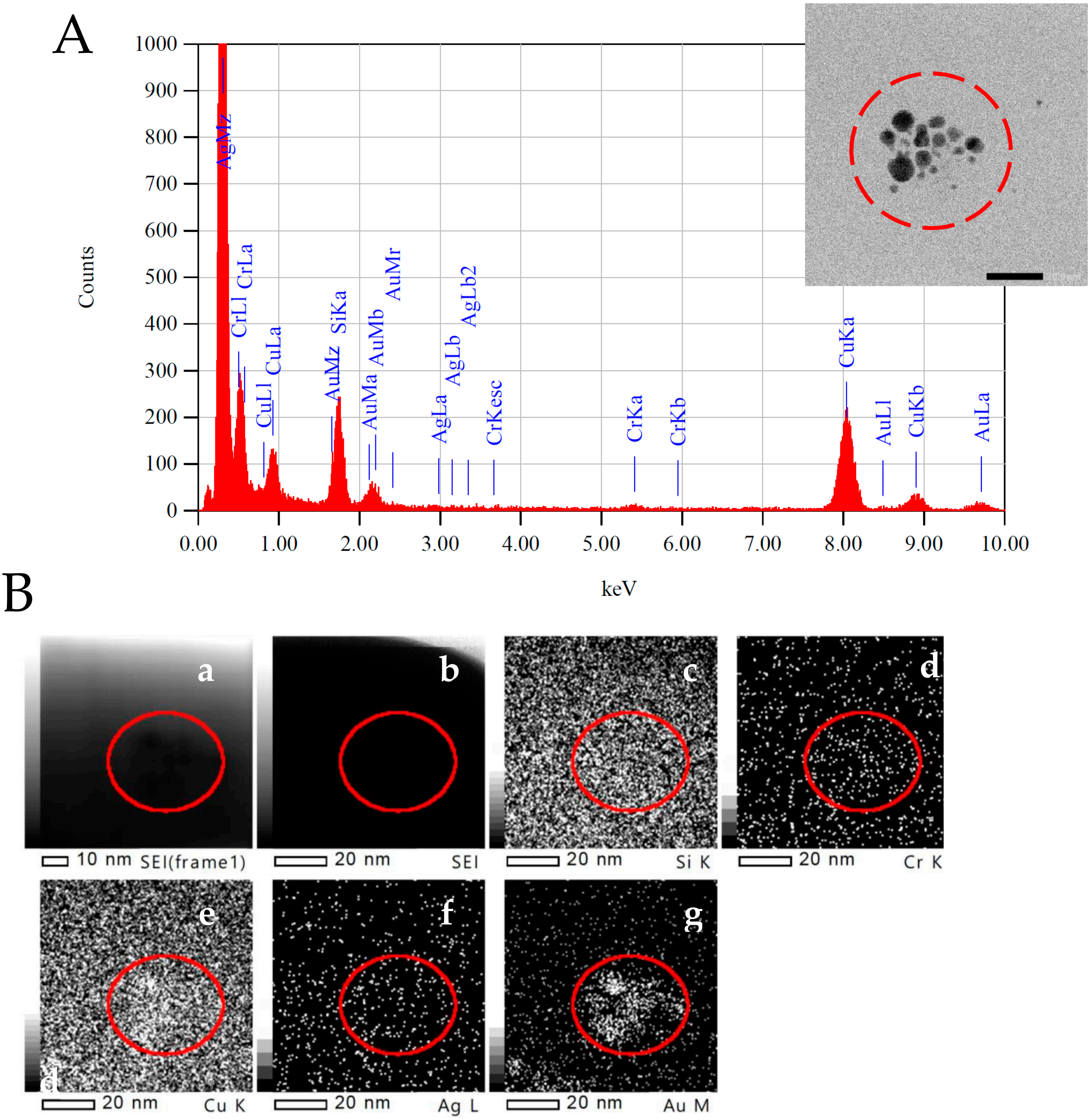

Figure S5. EDS analysis of field with NPs for 80:20 (area designed in image (inset) and **(B)** corresponding elemental mapping including (a) (b) carbon overlays, (c) Si, (d) Cr, (e) Cu, (f) Ag, and (g) Au. Note: SM-3, B-f (Ag) and SM-3B-g (Au) are also represented in Figure 7. It should be noted that chromium is a common impurity in mined glaze ingredients and the TEM grid is copper, so both are expected to be present.

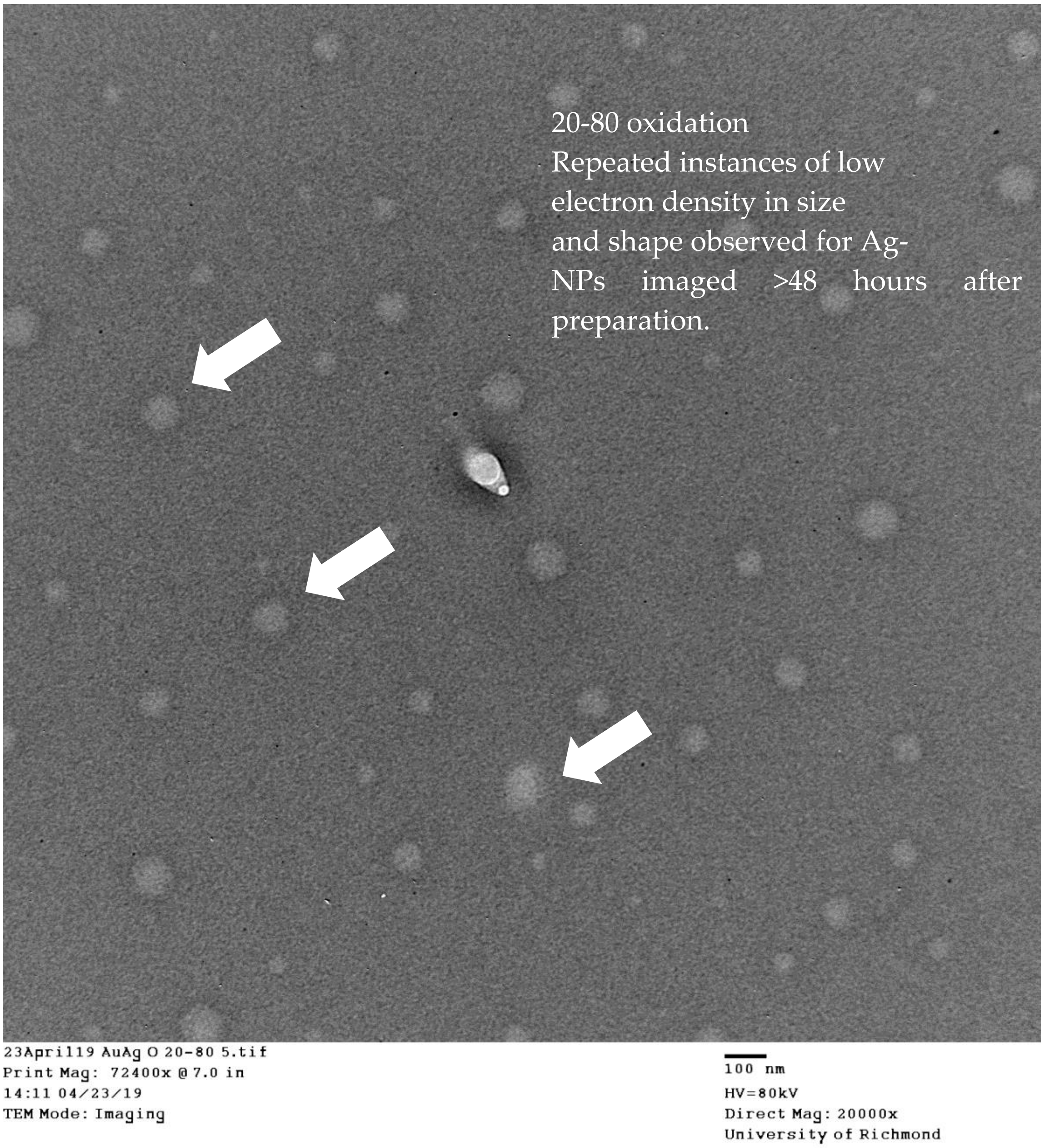

Figure S6. TEM imaging for 20:80 reduction sample showing low electron density holes and the corresponding absence of high electron density Ag NPs for a sample that was not immediately imaged.

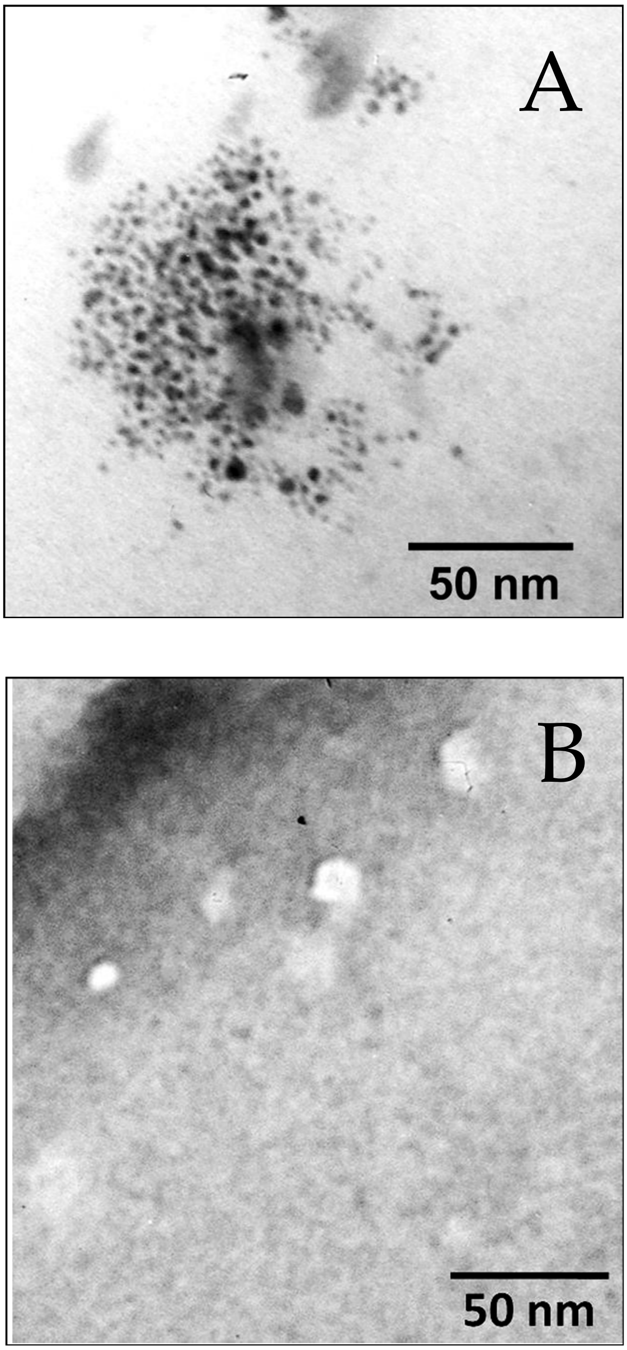

Figure S7. TEM imaging for 0:100 reduction sample **(A)** as prepared showing presence of Ag-NPs versus **(B)** the same sample imaged > 48 hours after being prepared and illustrating the absence of Ag-NPs (presence of low electron density holes with same dimensions as previously imaged Ag-NPs).
